# Factors Associated with Long-Term Prostate Cancer Survival after Palliative Radiotherapy to a Bone Metastasis and Contemporary Palliative Systemic Therapy: A Retrospective, Population-Based Study

**DOI:** 10.3390/curroncol30060420

**Published:** 2023-06-09

**Authors:** Bindu Venugopal, Shaheer Shahhat, James Beck, Nikesh Hanumanthappa, Aldrich D. Ong, Arbind Dubey, Rashmi Koul, Bashir Bashir, Amitava Chowdhury, Gokulan Sivananthan, Julian Oliver Kim

**Affiliations:** 1 Section of Radiation Oncology, Department of Radiology, Max Rady Faculty of Health Sciences, University of Manitoba, Winnipeg, MB R3E 0V9, Canada; 2 Department of Radiation Oncology, Kidwai Memorial Institute of Oncology, Bangalore 560029, India; 3 Department of Radiation Oncology, Western University, London, ON N6A 5W9, Canada; 4 Department of Medical Physics, CancerCare Manitoba, Winnipeg, MB R3E 0V9, Canada; 5 Department of Radiation Oncology, Kokilaben Dhirubhai Ambani Hospital and Medical Research Institute, Mumbai 400053, India; 6 CancerCare Manitoba Research Institute, CancerCare Manitoba, Winnipeg, MB R3E 0V9, Canada

**Keywords:** metastatic prostate cancer, bone metastases, palliative radiotherapy, survival intervals, prognostic factors

## Abstract

Background: Radiation therapy (RT) is an established palliative treatment for bone metastases; however, little is known about post-radiation survival and factors which impact it. The aim of this study was to assess a population-based sample of metastatic prostate cancer patients receiving palliative radiation therapy to bone metastases and contemporary palliative systemic therapy and identify factors that impact long-term survival. Materials/methods: This retrospective, population-based, cohort study assessed all prostate cancer patients receiving palliative RT for bone metastases at a Canadian provincial Cancer program during a contemporary time period. Baseline patient, disease, and treatment characteristics were extracted from the provincial medical physics databases and the electronic medical record. Post-RT Survival intervals were defined as the time interval from the first fraction of palliative RT to death from any cause or date of the last known follow-up. The median survival of the cohort was used to dichotomize the cohort into short- and long-term survivors following RT. Univariable and multivariable hazard regression analyses were performed to identify variables associated with post-RT survival. Results: From 1 January 2018 until 31 December 2019, 545 palliative RT courses for bone metastases were delivered to *n* = 274 metastatic prostate cancer patients with a median age of 76 yrs (Interquartile range (IQR) 39–83) and a median follow-up of 10.6 months (range 0.2 to 47.9). The median survival of the cohort was 10.6 months (IQR 3.5–25 months). The ECOG performance status of the whole cohort was ≤2 in *n* = 200 (73%) and 3–4 in *n* = 67 (24.5%). The most commonly treated sites of bone metastasis were the pelvis and lower extremities *n* = 130 (47.4%), skull and spine *n* = 114 (41.6%), and chest and upper extremities *n* = 30 (10.9%). Most patients had CHAARTED high volume disease *n* = 239 (87.2%). On multivariable hazard regression analysis, an ECOG performance status of 3–4 (*p* = 0.02), CHAARTED high volume disease burden (*p* = 0.023), and non-receipt of systemic therapy (*p* = 0.006) were significantly associated with worse post-RT survival. Conclusion: Amongst metastatic prostate cancer patients treated with palliative radiotherapy to bone metastases and modern palliative systemic therapies, ECOG performance status, CHAARTED metastatic disease burden, and type of first-line palliative systemic therapy were significantly associated with post-RT survival durations.

## 1. Introduction

Prostate cancer is the most common cancer amongst males in Canada [1]. Prostate cancer has an affinity for osseous tissue, making it the most frequent site of metastases [2]. It is estimated that 85–100% of the patients who die from prostate cancer harbor bone metastases [3]. The most common sequalae of bone metastases from prostate cancer are pain (35–45%), pathological fracture (14–22%), and spinal cord compression (3–7%) [4,5,6,7,8]. Radiation therapy with single and multiple fractions have demonstrated similar efficacy and are widely employed to palliate symptoms of bone metastases from prostate cancer [9,10,11]. Although radiation therapy is an established modality for treating bone metastases, little is known about post-radiation survival and the factors which impact it. The primary aim of this study was to assess a cohort of metastatic prostate cancer patients receiving palliative radiation therapy to bone metastases and identify factors that impact long-term survival.

The backbone of palliative systemic therapy for prostate cancer is androgen deprivation therapy (ADT) achieved either with orchidectomy or medically with LHRH agonists [12]. Various palliative systemic therapy options for prostate cancer in addition to ADT have been introduced over the last decade [13] which address castration-resistant disease including abiraterone and androgen receptor-axis-targeted therapies (ARAT) [14,15], chemotherapy such as taxanes, and Radium-223 [13,16]. Bisphosphonates and agents which inhibit the receptor activator of nuclear factor-κB ligand (RANKL) [17] are useful adjuncts to the aforementioned systemic therapies which can prevent additional skeletal related events [7,18].

Many prostate cancer patients with metastatic disease receive palliative radiotherapy to bone metastases in the setting of increasing use of novel systemic therapies. However, little is known regarding the impact of modern palliative systemic therapies on survival post completion of palliative RT. Descriptions of survival intervals and factors influencing survival following palliative RT for prostate cancer bone metastases would be of clinical utility for radiation oncologists so to help them decide if intensification of palliative RT (i.e., hypofractionated RT or stereotactic body radiotherapy (SBRT)) may be warranted for cases for whom the survival interval is expected to be prolonged. This study, therefore, aims to describe the survival trajectories of a large population-based cohort of prostate cancer patients receiving contemporary palliative systemic treatments after the receipt of palliative RT to bone metastases and identify factors associated with prolonged survival.

## 2. Materials and Methods

This retrospective, population-based cohort study assessed all prostate cancer patients undergoing palliative radiotherapy for bone metastases at CancerCare Manitoba (CCMB), which is the publicly funded, sole source cancer treatment agency with a catchment of 1.4 million persons living in the Canadian province of Manitoba and the Territory of Nunavut. This study was approved by the University of Manitoba Health Research Ethics Board (approval #: HS20808) and the CCMB Research Resource Impact Committee (approval #: 2017-020).

All metastatic prostate cancer patients treated with palliative radiation therapy (RT) for a bone metastasis between 1 January 2018 to 31 December 2019 at CCMB were identified using the CCMB medical physics database. Individual patient characteristics were obtained from the CCMB electronic medical record (EMR). Patient, treatment, and disease factors were extracted and tabulated, dichotomized by the median survival of the cohort. Variables collected include the following: age at time of RT, year of RT, site of bone metastases, Charlson comorbidity index, Eastern Co-operative Oncology Group (ECOG) performance status, and complicated versus uncomplicated bone metastases as defined by Cheon et al. [19]. Complicated bone metastases, for the purpose of this study, were defined as painful metastases associated with existing or impending pathological fracture, spinal cord compression, or cauda equina compression. Volume of metastatic disease was classified using the CHAARTED trial definition as high-volume disease if a patient had ≥ 4 bone metastases, >1 metastasis outside the axial skeleton or pelvis, and/or visceral metastases [13]. All other metastatic disease distributions were classified as low volume disease. Other variables extracted included number of bone metastases, visceral metastases, and date of castration resistance. Treatment characteristics such as RT dose-fractionation schedule, palliative RT to a bone lesion other than the index lesion, repeat palliative RT to the index lesion, prior management of prostate primary, bisphosphonate, first line, second line, and third line palliative systemic therapy were also extracted from the EMR. The post-RT survival interval was defined as date of first fraction of RT to date of death (of any cause) or date of last follow-up.

## 3. Statistical Considerations

The database was frozen for analysis on the 15 March 2022. The median post-RT survival was calculated for the whole cohort in order to dichotomize the cohort into short-term and long-term survival groups. The data were tabulated by survival group, and the differences in distributions of variables across survival groups were assessed using standard statistical tests (chi-square and student *t*-test). Baseline patient, treatment, and disease variables were assessed for their association with death from any cause using univariable hazard regression. A multivariable hazard regression model was built and variables with univariable *p*-values of <0.2 were added to the model using a forward, stepwise selection process. Actuarial Kaplan–Meier survival curves were generated for visualization of the survival estimates of the cohort overall and stratified by variables of interest arising from the univariable and multivariable hazard regression analysis. The Log–Rank test was used to test for differences in survival estimates by subgroups of interest. For purposes of this analysis, *p*-values < 0.05 were considered statistically significant. All statistical analyses were carried out using the STATA statistical software, version 15 (Statacorp, College Station, TX, USA).

## 4. Results

From 1 January 2018 to 31 December 2019, 545 palliative RT courses for bone metastases were delivered to 274 metastatic prostate cancer patients at CCMB. Of the 274 patients included in the analysis, the median follow-up was 10.6 months (range 0.2 to 47.9 months). At the time of analysis, the majority of the cohort had died (*n* = 228 (83.2%)). The median survival of the cohort was 10.6 (IQR, 3.5–25.0) months. The cohort was dichotomized using the median survival time into short-term survivors (survival time < 10.6 months) and long-term survivors (survival time ≥ 10.6 months). Amongst short-term survivors (*n* = 137), the median survival was 3.5 months (IQR 1.8 to 6.3 months), and amongst long-term survivors (*n* = 137), the median survival was 25.0 months (IQR 17.5 to 32.1 months). 

### 4.1. Patient Characteristics

The median age at the time of RT for the overall cohort was 76 years (IQR = 69–83), which did not differ significantly by survival cohort: 76 (IQR 69–83) vs. 76 (IQR 70–82), *p* = 0.99, for short-term and long-term survival groups, respectively. The distribution of baseline patient and disease characteristics are tabulated in Table 1. The Charlson index of the entire cohort was 0–1 in *n* = 185 (67.5%), 2–3 in *n* = 68 (24.8%), and ≥4 in *n* = 21 (7.7%), and the distribution did not differ significantly between the two survival groups (*p* = 0.52). The ECOG performance status for the whole cohort was ≤2 in *n* = 200 (73%) and 3–4 in *n* = 67 (24.5%). In the short-term survival group, the proportion with ECOG 3 or 4 was significantly greater compared to the long-term survival group: 32.6% (short-term survival group) vs. 17.8% (long-term survival group), *p* = 0.005.

### 4.2. Disease Characteristics

The anatomic sites of bone metastasis most commonly treated with RT were the pelvis and lower extremities *n* = 130 (47.4%), the skull and spine *n* = 114 (41.6%), and the chest and upper extremities *n* = 30 (10.9%), and the distribution was not significantly different between long- and short-term survivors (*p* = 0.72). Complicated bone metastases were seen in *n* = 98 (35.8%) of the whole cohort, of which *n* = 50 (36.5%) were in the short-term survival group and *n* = 48 (35.8%) were in the long-term survival group (*p* = 0.80). Sixty (21.9%) patients in the whole cohort had visceral metastases at the time of RT; *n* = 35 (28.2%) were in the short-term survival group, and *n* = 25 (19.8%) were in the long-term survival group, *p* = 0.12. The number of bone metastases was ≥4 in *n* = 231 (84.3%) of the whole cohort, *n* = 122 (89.1%) in the short-term group, and *n* = 109 (79.6%) in the long-term group (*p* = 0.136). Most patients had CHAARTED high volume disease, *n* = 239 (87.2%), while a minority had CHAARTED low volume disease, *n* = 35 (12.8%) in the entire cohort. In the short-term group, *n* = 124 (90.5%) had CHAARTED high-volume disease while a lower proportion of CHAARTED high-volume disease was in the long-term survival group *n* = 115 (83.9%), *p* = 0.10. Castration resistance with second line therapy was see in *n* = 151 (55.1%) of the whole cohort, with *n* = 69 (50.4%) in the short-term survival group and *n* = 82 (59.9%) in the long-term survival group, (*p* = 0.16). 

### 4.3. Treatment Characteristics

The treatment characteristics are summarized in Table 2. Single fraction palliative radiation therapy (8 Gy/1#) predominated with *n* = 210 (76.6%) of the entire cohort and *n* = 108 (78.8%) of short-term and *n* = 102 (74.5%) of long-term groups receiving it (*p* = 0.39). One hundred and fifty patients (54.7%) received palliative RT to bone metastases other than the index lesion. This was seen often amongst long-term survivors, *n* = 88 (64.2%) vs. short-term survivors *n* = 62 (45.3%), (*p* = 0.002). The number of patients who received repeat radiation to the index lesion was *n* = 22 (8%) for the whole cohort, which did not differ significantly between survival groups: *n* = 15 (11%) short-term vs. *n* = 7 (5.1%) long-term (*p* = 0.075).

Out of the *n* = 99 (36.1%) patients who received treatment to the prostate primary, the distribution of the patients was equal in the long- and short-term groups *n* = 49 (35.5%) and *n* = 49 (35.5%), respectively. The most common type of treatment to the prostate primary was radical radiation therapy in 32 (11.7%) cases, with non-significant distribution across the two groups: 21 (15.3%) vs. 18 (13.1%) in short vs. long, respectively, (*p* = 0.27). Other antecedent treatments to the prostrate included prostatectomy *n* = 27 (9.9%), trans-urethral resection of prostate *n* = 7 (2.6%), primary brachytherapy *n* = 8 (2.9%), surgery followed by salvage radiation therapy *n* = 17 (6.2%), and high frequency ultrasound *n* = 1 (0.4%). 

First line systemic therapy was employed widely, *n* = 269 (98.2%), consisting of LHRH alone *n* = 212 (77.4%), LHRH plus taxane *n* = 30 (11%), LHRH plus abiraterone or ARAT *n* = 17 (6.2%), and bicalutamide alone *n* = 10 (3.6%). In the first line, 4 (2.9%) patients in the short-term group and 1 (0.7%) patient in the long-term group did not receive systemic therapy, (*p* = 0.04). Bisphosphonates were commonly utilized in the study cohort with *n* = 192 (70.1%) receiving them, consisting of *n* = 103 (75.2%) short-term survivors vs. *n* = 89 (65%) long-term survivors, (*p* = 0.12). Of the patients treated with LHRH plus abiraterone or ARAT, a greater proportion came from the long-term survival group (*n* = 14 (10.2%)) vs. the short-term survival group (*n* = 3 (2.2%), *p* = 0.04). Second line systemic therapy was utilized by *n* = 179 (65.3%) of the cohort, consisting of abiraterone or ARAT *n* = 112 (62.6%), LHRH agonist plus bicalutamide *n* = 43 (24%), LHRH agonist plus a taxane *n* = 20 (11.2%), and LHRH agonist plus Radium-223 *n* = 4 (2.2%). There were no differences in the distribution of second line palliative systemic therapy between the two groups (*p* = 0.83). Third line systemic therapy was utilized by *n* = 95 (34.7%) of the whole cohort, of which taxanes were employed in *n* = 72 (75.8%) cases with no difference in distribution of the third line therapies across the two groups (*p* = 0.77). 

### 4.4. Survival Characteristics

Survival intervals of the cohort dichotomized by baseline characteristics identified as statistically significant in the multivariable hazard regression are reported below. Patients with an ECOG performance status of 0–2 survived a median of 13.2 months (IQR 4.4 to 26.7) while patients with an ECOG of 3–4 survived a median of 4.8 months (IQR 1.6 to 17). Patients with CHAARTED low volume disease survived a median of 16.8 months (IQR 3.5 to 29.2) while those with high volume disease survived a median of 9.8 months (IQR 3.5 to 24.9). Ordered by first line palliative systemic therapy type, median survival times were as follows: LHRH only (9.7 months, IQR 3.5 to 24.9); LHRH plus taxane (10.2 months, IQR 4.3 to 24.9); LHRH Plus Abiraterone or ARAT (21.5 months, IQR 16.0 to 31.9); Bicalutamide alone (12.1 months, IQR 5.5 to 22.2); and no palliative systemic therapy (1.6 months, IQR 0.7 to 3.0).

**Figure 1:**
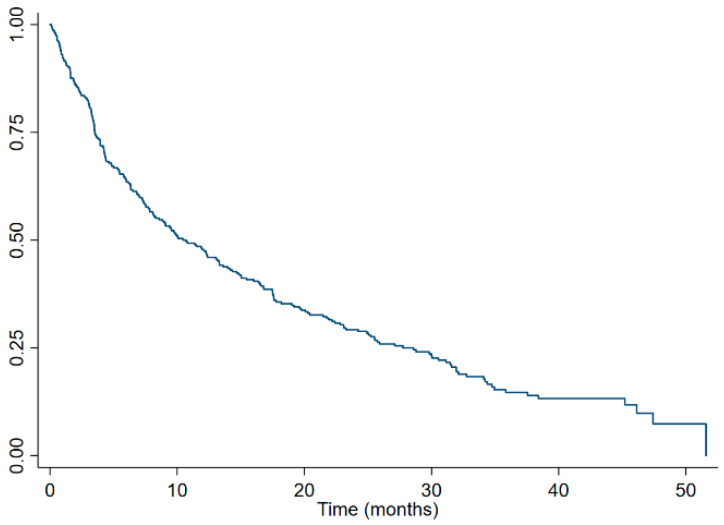
Kaplan–Meier survival estimates of the whole cohort.

**Figure 2:**
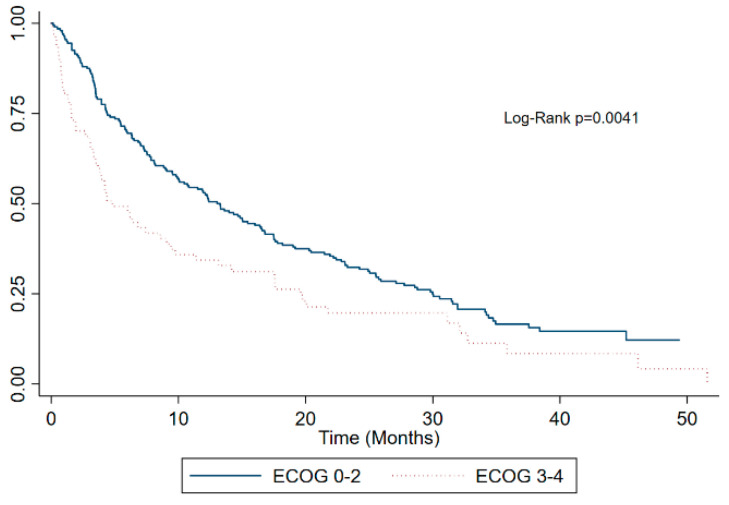
Kaplan–Meier survival estimates of the whole cohort by ECOG performance status.

**Figure 3:**
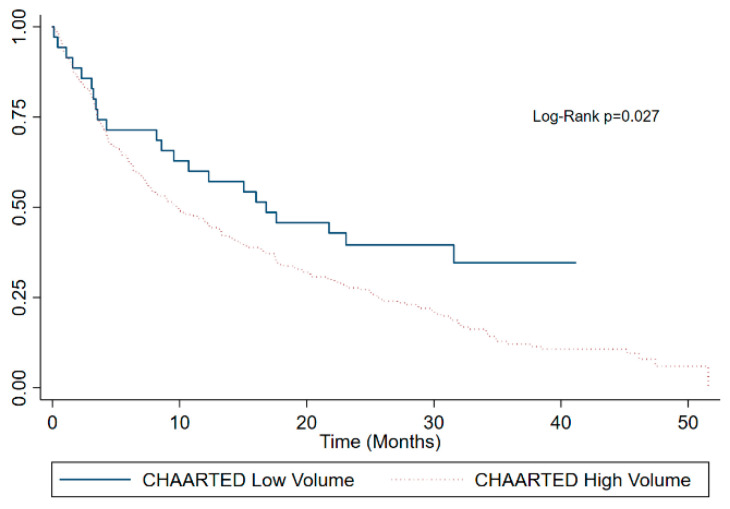
Kaplan–Meier survival estimates by CHAARTED classification of metastatic disease burden.

**Figure 4:**
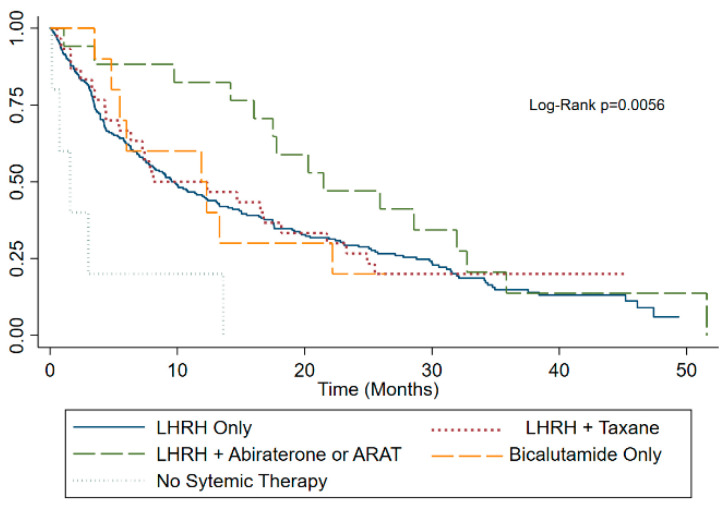
Kaplan–Meier survival estimate by first line palliative systemic therapy.

Kaplan–Meier survival estimates of the whole cohort (Figure 1) and stratified by the ECOG performance status (Figure 2), CHAARTED disease burden category (Figure 3), and first line palliative systemic therapy received (Figure 4) are presented below.

### 4.5. Hazard Regression Analysis

The results of the univariable and multivariable analyses are presented in Table 3 and Table 4, respectively. On univariable hazard regression analysis, Charlson scores of ≥4 (*p* = 0.014), an ECOG performance status of 3–4 (*p* = 0.004), CHAARTED high volume disease (*p* = 0.029), and non-receipt of palliative systemic therapy (*p* = 0.002) were significantly associated with worse overall survival. 

In the multivariable hazard regression analysis, an ECOG performance status of 3–4 (*p* = 0.02), CHAARTED high volume disease burden (*p* = 0.023), and non-receipt of systemic therapy (*p* = 0.006) were significantly associated with short-term survival. 

Median overall survival durations and IQRs by subgroups of interest identified in the multivariable hazard regression as being associated with survival are tabulated in Table 5.

## 5. Discussion

Clinicians prescribing palliative radiotherapy must carefully consider who should be treated with simple palliative radiotherapy techniques/doses (such as 8 Gy in 1 fraction with two-field techniques) versus more complex, intensified palliative treatments (i.e., SBRT). The rationale for more intensified palliative radiotherapy (SBRT) is predicated on reasonable expectations that candidates for such techniques will survive long enough to benefit from them and therefore justify their increased costs and workloads over standard techniques. 

In recent history, the management of metastatic prostate cancer patients has evolved considerably with the introduction of a number of novel palliative systemic treatment options available including ARATs, abiraterone [20], taxanes [21], and Radium-223 [16] which have all demonstrated improved survival outcomes for prostate cancer patients. Furthermore, in late 2018, the results of the STAMPEDE Arm H were published demonstrating a survival advantage amongst metastatic prostate cancer patients with low metastatic burdens treated with RT to the prostate primary (including SBRT) [22]. Over the last several years, stereotactic ablative radiotherapy techniques for prostate cancer patients with bony metastases disease have become more readily accessible to radiation oncologists with several phase 3 randomized clinical trials currently underway (ClinicalTrials.gov (URL accessed on 6 June 2023) Identifiers: NCT03784755, NCT02685397) assessing the role of SBRT for metastatic prostate cancer patients with castration-sensitive and castration-resistant diseases. Reported randomized phase 2 studies, conducted on cohorts that were comprised of high proportions of prostate cancer patients, have also suggested that SBRT for bone metastases may improve survival outcomes and may afford superior local control over standard palliative RT [23,24]. This has resulted in cautious optimism for the use of SBRT in the metastatic prostate cancer milieu in routine clinical practice (outside of clinical trials). Therefore, survival outcomes of metastatic prostate cancer patients are potentially more heterogenous than ever, given the variety of treatment options available for them.

The prognostication of survival of prostate cancer patients can be a challenging task. In this population-based, retrospective cohort study of metastatic prostate cancer patients, which included both castration-sensitive and castration-resistant cases undergoing palliative radiotherapy to bone metastases, we found that prognosis was highly heterogenous. Those with the shortest survival were observed to have a poor performance status (ECOG 3–4, adjusted HR = 1.45, *p* = 0.02), to have CHAARTED High Volume disease at the time of RT (adjusted HR, *p* = 0.023), and did not received any palliative systemic therapy (adjusted HR = 3.61, *p* = 0.006). Thus, patients with these characteristics would be unlikely to benefit from intensified therapy for their bone metastases. The CHAARTED high metastatic burden variable has been associated with a shorter survival interval from prostate cancer diagnosis to death with a median survival of 103 months for low volume disease vs. 62.7 month for high volume disease [25]. Our study’s findings confirm that the CHAARTED low-versus-high disease burden classification maintains prognostic significance for the time interval spanning from the time of RT to death. The ECOG performance status has been demonstrated to be prognostic for prostate cancer patients in various settings, including for mCRPC with first line chemotherapy [26,27]. A systematic review on the prognostic value of the ECOG and the Gleason scores in castration-resistant prostate cancer found that an ECOG score of more than 2 was associated with a significantly increased risk of mortality [28]. The studies assessing systemic therapy with taxanes, abiraterone, or ARAT in the metastatic setting usually included patients with an ECOG of 0–1 and excluded patients with worse ECOG performance status. In our real-world study, we found that an ECOG performance score of ≥3 significantly increased the risk of death following palliative radiotherapy for bone metastases on multivariate analysis. The variation in our reported survival intervals by first line systemic therapy likely represents, to a considerable extent, the selection bias of prescribing clinicians and should therefore be interpreted with caution. Not surprisingly, the presence of any of the three prognostic variables would also render a prostate cancer patient ineligible from any of the randomized trials currently underway investigating SBRT for bone metastases of prostate cancer patients with metastatic disease including PLATON (ClinicalTrials.gov Identifier: NCT03784755) and the Group-Q PCS-IX study (ClinicalTrials.gov Identifier: NCT02685397), highlighting the need for real-world data. The findings of this study, therefore, validate the use of the selection criteria of the studies in the metastatic prostate cancer milieu. A review of the assessments of prognostic factors associated with survival following the receipt of palliative radiotherapy for a bone metastasis reported in the literature, including from randomized controlled trials, have typically included heterogenous groups of patients with assortments of different primary cancer types in the same prognostic model [29,30]. We have not found any published evaluations of prognostic models built specifically using prostate cancer patients following a receipt of palliative RT for bone metastasis. 

In this study, we found that a real-world, population-based cohort of metastatic prostate cancer patients survived a median of 10.6 months (IQR, 3.5–25.0) following the receipt of their first course of palliative radiotherapy. Our survival outcomes mirrored the results of a similar study by the British Columbia Cancer Agency (BCCA), during the time period just prior to the current study, which reported a median survival time of prostate cancer patients following the receipt of their first course of palliative RT of 8.6 months [31]. In the BCCA study, a gradual time-trend of improvement in median survival of metastatic prostate cancer patients was observed from 8.2 to 9.4 months over a time period spanning 12 years ending in 2015. During our study period (2018–2019) the use of contemporary palliative systemic treatments (ie., ARATs, abiraterone, taxanes, and Radium-223) was rare (6.2%) in the first line setting but rose considerably to 49.6% of the entire cohort in the second line setting (following disease progression to castration-resistant disease after LHRH agonists alone). During the years subsequent to this study (i.e., 2020 to present), the use of these novel agents has been expanded in our jurisdiction as first line palliative systemic therapy for patients with metastatic, castration-sensitive disease which would be expected to modestly improve the median survival amongst metastatic prostate cancer patients when these data are examined again in the future. 

### Study Limitations

The main limitation of this study is the retrospective design which makes it prone to selection bias issues especially the choice of palliative systemic therapies. Thus, survival estimates by first line palliative systemic therapy are not only a result of the type of therapy received but also inherently reflect the selection, by clinicians, of robust patients who are fit enough to undergo intensified systemic treatments over LHRH agonists alone. We have, therefore, attempted to mitigate some of the impact of clinician selection bias by including a large population-based sample annotated with characteristics such as age, performance status, and Charlson index in the multivariable models assessing survival. Despite this, we concede that there is likely still residual unaccounted for confounders which remain. 

## 6. Conclusions

Amongst metastatic prostate cancer patients treated with palliative radiotherapy to bone metastases in the context of modern palliative systemic therapies, ECOG performance status, CHAARTED burden of metastatic disease, and type of first line palliative systemic therapy were significantly associated with post-RT survival durations. 

## Figures and Tables

**Table 1:** Baseline patient and disease characteristics of the cohort.

Variable	Whole Cohort (*n* = 274)	Short Term Survivors (*n* = 137)	Long Term Survivors (*n* = 137)	*p*-Value
**Age at time of RT (median, range)**	76 (69–83)	76 (69–83)	76 (70–82)	0.99
**Year of RT** 2018	137 (50%)	69 (50.4%)	68 (49.6%)	0.904
2019	137 (50%)	68 (49.6%)	69 (50.4%)	
**Site of bone metastasis RT** Skull/Spine	114 (41.6%)	57 (41.6%)	57 (41.6%)	0.720
Chest and upper extremity	30 (10.9%)	17 (12.4%)	13 (9.5%)	
Pelvis and lower extremity	130 (47.4%)	63 (46.0%)	67 (48.9%)	
**Multifraction Radiotherapy** Yes	64 (23.4%)	29 (21.17%0)	35 (25.55%)	0.392
No	210 (76.6%)	108 (78.8%)	102 (74.5%)	
**Charlson Index** 0–1	185 (67.5%)	91 (66.4%)	94 (68.6%)	0.523
2–3	68 (24.8%)	33 (24.1%)	35 (25.6%)	
≥4	21 (7.7%)	13 (9.5%)	8 (5.8%)	
**ECOG Performance Status** 0–2	200 (73.0%)	89 (67.4%)	111 (82.2%)	0.005
3–4	67 (24.5%)	43 (32.6%)	24 (17.8%)	
**Complicated bone metastases** Yes	98 (35.8%)	50 (36.5%)	48 (35.0%)	0.801
No	176 (64.2%)	87 (63.5%)	89 (64.2%)	
**Visceral metastases at RT** Yes	60 (21.9%)	35 (28.2%)	25 (19.8%)	0.121
No	190 (69.3%)	89 (71.8%)	101 (80.2%)	
**Number of bone metastases** 1–3	43 (15.7%)	15 (11.0%)	28 (20.4%)	0.136
≥4	231 (84.3%)	122 (89.0%)	109 (79.6%)	
**CHAARTED Disease Burden** Low	35 (12.8%)	13 (9.5%)	22 (16.1%)	0.103
High	239 (87.2%)	124 (90.5%)	115 (83.9%)	
**Castration resistance with 2nd** **line systemic therapy** Yes	151 (55.1%)	69 (50.4%)	82 (59.9%)	0.160
No	28 (10.2%)	18 (13.1%)	10 (7.3%)	

Abbreviations: RT—radiation therapy, ECOG—Eastern Cooperative Oncology Group, CHAARTED—Chemohormonal Therapy Versus Androgen Ablation Randomized Trial for Extensive Disease in Prostate Cancer.

**Table 2:** Treatment characteristics of the cohort.

Variable	Whole Cohort (*n* = 274)	Short Term Survivors (*n* = 137)	Long Term Survivors (*n* = 137)	*p*-Value
**Palliative RT to bone metastases** **other than index lesion** Yes	150 (54.7%)	62 (45.3%)	88 (64.2%)	0.002
No	124 (45.3%)	75 (54.7%)	49 (35.8%)	
**Repeat Palliative RT to index lesion** Yes	22 (8.0%)	15 (11.0%)	7 (5.1%)	0.075
No	252 (92.0%)	122 (89.1%)	130 (94.9%)	
**Prior Management of prostate primary** None	175 (63.9%)	89 (65.0%)	86 (62.8%)	0.278
Prostatectomy	27 (9.9%)	13 (9.5%)	14 (10.2%)	
Radical radiation therapy	32 (11.7%)	21 (15.3%)	18 (13.1%)	
Transuretheral resection of prostate only	7 (2.6%)	2 (1.5%)	5 (3.7%)	
Primary brachytherapy	8 (2.9%)	6 (4.4%)	2 (1.5%)	
Prostatectomy followed by salvage RT	17 (6.2%)	5 (3.7%)	12 (8.8%)	
High frequency ultrasound	1 (0.4%)	1 (0.7%)	0 (0%)	
**Bisphosphonate use** No	192 (70.1%)	103 (75.2%)	89 (65.0%)	0.128
Yes	81 (29.6%)	34 (24.8%)	47 (34.3%)	
**First line systemic therapy** LHRH agonist alone	212 (77.4%)	111 (81.0%)	101 (73.7%)	0.044
LHRH and Taxane	30 (10.9%)	15 (11.0%)	15 (11.0%)	
LHRH and abiraterone or ARAT	17 (6.2%)	3 (2.2%)	14 (10.2%)	
Bicalutamide alone	10 (3.6%)	4 (2.9%)	6 (4.4%)	
No systemic therapy	5 (1.8%)	4 (2.9%)	1 (0.7%)	
**Second line systemic therapy** LHRH and Taxane	20 (7.3%)	10 (7.3%)	10 (7.3%)	0.83
LHRH and Abiraterone or ARAT	112 (40.9%)	54 (39.4%)	58 (42.3%)	
LHRH and bicalutamide	43 (15.7%)	22 (16.1%)	21 (15.3%)	
LHRH and Radium-223	4 (1.5%)	1 (0.7%)	3 (2.2)	
No second line systemic therapy	95 (34.7%)	50 (36.5%)	45 (32.9%)	
**Third line systemic therapy** LHRH and Taxane	72 (26.3%)	35 (25.6%)	37 (27.0%)	0.774
LHRH and Abiraterone or ARAT	17 (6.2%)	10 (7.3%)	7 (5.1%)	
LHRH and Radium-223	5 (1.8%)	3 (2.2%)	2 (1.5%)	
LHRH and Rupcaparib	1 (0.4%)	0 (0%)	1 (0.7%)	
No third line systemic therapy	179 (65.3%)	89 (64.96%)	90 (65.7%)	

Abbreviations: RT—radiation therapy, ARAT—androgen receptor axis-targeted therapy, LHRH—luteinizing hormone releasing hormone—CHAARTED Chemohormonal Therapy Versus Androgen Ablation Randomized Trial for Extensive Disease in Prostate Cancer.

**Table 3:** Univariable hazard regression analysis for all-cause mortality.

Variable	Hazard Ratio	*p* Value
**Age at the time of RT** <76 years	Ref	Ref
≥76 years	1.23	0.122
**Year of RT** 2018	Ref	Ref
2019	1.05	0.699
**Site of RT** Skull	Ref	Ref
Upper extremity	5.09	0.112
Chest	5.51	0.114
Spine	5.28	0.098
Pelvis	4.56	0.131
Lower extremity	3.52	0.219
**Receipt of Multi-fraction RT**	0.81	0.215
**Charlson Index** 0–1	Ref	Ref
2–3	1.2	0.221
≥4	1.8	0.014
**ECOG Performance status** 0–2	Ref	Ref
3–4	1.54	0.004
**Complicated bone metastases** No	Ref	Ref
Yes	1.18	0.211
**Visceral metastases** No	Ref	Ref
Yes	1.30	0.102
**Number of bone metastases** 1–3	Ref	Ref
≥4	1.81	0.031
**CHAARTED Disease Burden** Low	Ref	Ref
High	1.62	0.029
**Bisphosphonate use** No	Ref	Ref
Yes	0.80	0.128
**First Line Systemic Therapy**		
LHRH agonist alone	Ref	Ref
LHRH and taxane	0.93	0.773
LHRH with abiraterone or ARAT	0.65	0.126
Bicalutamide alone	1.03	0.915
No systemic therapy	4.16	0.002

Abbreviations: RT—radiation therapy, ARAT—androgen receptor axis-targeted therapy, LHRH—luteinizing hormone releasing hormone, CHAARTED—Chemohormonal Therapy Versus Androgen Ablation Randomized Trial for Extensive Disease in Prostate Cancer.

**Table 4:** Multivariable hazard regression analysis for all-cause mortality.

Factor	Hazard Ratio	*p* Value
**Age at the time of RT** <76 years	Ref	Ref
≥76 years	1.01	0.146
**Charlson Index** 0–1	Ref	Ref
2–3	1.18	0.303
≥4	1.46	0.125
**ECOG Performance Status** 0–2	Ref	Ref
3–4	1.45	0.02
**CHAARTED Disease Burden** Low	Ref	Ref
High	1.71	0.023
**First Line Systemic Therapy**		
LHRH agonist alone	Ref	Ref
LHRH and taxane	1.06	0.777
LHRH with abiraterone or ARAT	0.63	0.111
Bicalutamide alone	0.907	0.8
No systemic therapy	3.61	0.006

Abbreviations: RT—radiation therapy, ECOG—Eastern Cooperative Oncology Group, ARAT—androgen receptor axis-targeted therapy, LHRH—luteinizing hormone releasing hormone, CHAARTED—Chemohormonal Therapy Versus Androgen Ablation Randomized Trial for Extensive Disease in Prostate Cancer.

**Table 5:** Survival strata by prognostic factors of interest identified from the multivariable analysis.

Variable	Median Post-RT Survival in Months (IQR)
**ECOG Performance Status**	
0	25.5 (15.4–34.3)
1	12.1 (4.6–24.3)
2	10.4 (3.4–25.9)
3	6.1 (1.9–19.6)
4	2.2 (0.6–8.7)
**CHAARTED Disease Burden**	
Low	16.8 (3.5–29.1)
High	9.8 (3.5–25)
**First line systemic therapy Received**	
LHRH agonist alone	9.6 (3.5–24.9)
LHRH and taxane	10.2 (4.3–25)
LHRH and abiraterone or ARAT	21.5 (16–32)
Bicalutamide alone	12.1 (5.5–22.2)
No systemic therapy	1.6 (0.7–3)

Abbreviations: RT—radiation therapy, ECOG—Eastern Cooperative Oncology Group, ARAT—androgen receptor axis-targeted therapy, LHRH—leutinizing hormone releasing hormone, CHAARTED—Chemohormonal Therapy Versus Androgen Ablation Randomized Trial for Extensive Disease in Prostate Cancer.

## Data Availability

The data utilized in this study are available upon reasonable request to the corresponding author.
